# ZIF-8@Hydroxyapatite Composite as a High Potential Material for Prolonged Delivery of Agrochemicals

**DOI:** 10.1021/acsami.4c06016

**Published:** 2024-05-27

**Authors:** Samuel Morales-Cámara, Belén Parra-Torrejón, Antonio Rodríguez-Diéguez, José M. Delgado-López, Gloria B. Ramírez-Rodríguez, Sara Rojas

**Affiliations:** Department of Inorganic Chemistry, University of Granada, Av. Fuentenueva, s/n, 18071 Granada, Spain

**Keywords:** metal−organic frameworks, hydroxyapatite, agriculture, antibacterial, fertilizer

## Abstract

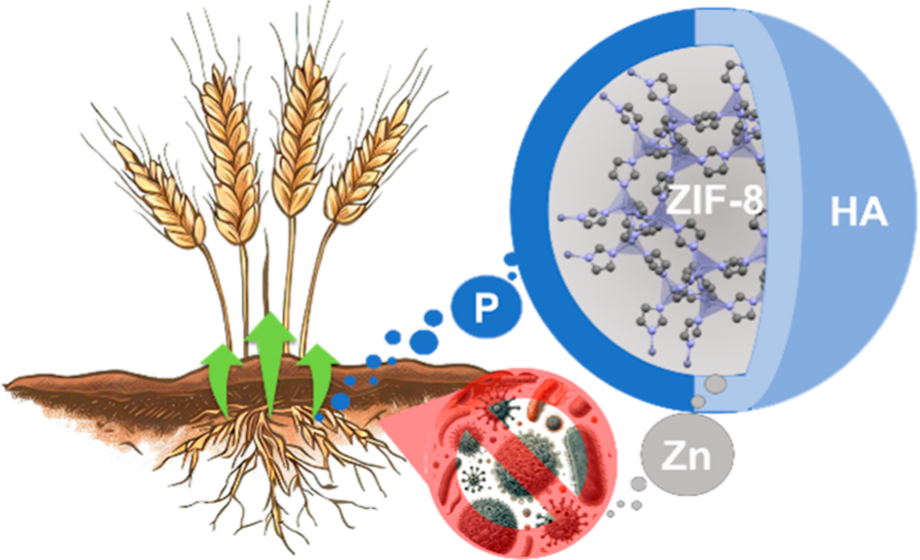

Although agrochemical practices can enhance agricultural productivity, their intensive application has resulted in the deterioration of ecosystems. Therefore, it is necessary to develop more efficient and less toxic methods against pests and infections while improving crop productivity. Moving toward sustainable development, in this work, we originally described the preparation of a composite (ZIF-8@HA) consisting of the coating of zeolitic-like metal–organic framework (MOF) ZIF-8 (based on Zn, an essential micronutrient in plants with antibacterial, antifungal, and antifouling properties) with hydroxyapatite (HA) nanoparticles (i.e., nanofertilizer). The interaction between the HA and ZIF-8 has been characterized through a combination of techniques, such as microscopic techniques, where the presence of a HA coating is demonstrated; or by analysis of the surface charge with a dramatic change in the Z-potential (from +18.7 ± 0.8 to −27.6 ± 0.7 mV for ZIF-8 and ZIF-8@HA, respectively). Interestingly, the interaction of HA with ZIF-8 delays the MOF degradation (from 4 h for pristine ZIF-8 to 168 h for HA-coated material), providing a slower and gradual release of zinc. After a comprehensive characterization, the potential combined fertilizer and bactericidal effect of ZIF-8@HA was investigated in wheat (*Triticum aestivum*) seeds and *Pseudomonas syringae* (*Ps*). ZIF-8@HA (7.3 ppm) demonstrated a great fertilizer effect, increasing shoot (9.4 %) and root length (27.1 %) of wheat seeds after 11 days at 25 °C under dark conditions, improving the results obtained with HA, ZIF-8, or ZnSO_4_ or even physically mixed constituents (HA + ZIF-8). It was also effective in the growth inhibition (>80 % of growth inhibition) of *Ps*, a vegetal pathogen causing considerable crop decline. Therefore, this work demonstrates the potential of MOF@HA composites and paves the way as a promising agrochemical with improved fertilizer and antibacterial properties.

## 1 Introduction

Excessive use of agrochemicals is degrading ecosystem quality (living beings, soils, and groundwaters), ultimately affecting human health. The major issues related with the extensive/excessive use of agrochemicals are (i) growing population (projected to be 9.7 billion in 2050),^1^ (ii) poor efficacy (between 10 and 75 % do not reach their target),^2,3^ (iii) high environmental impact (contaminating water, soil, and air),^4^ and (iv) the development of resistant straights.^5^ However, the use of agrochemicals (9.8 million tons of N-based fertilizers and 1.1 million tons of P-based fertilizers were used in agriculture across the European Union only in 2021)^6^ is crucial to combat pests and supply the lack of nutrients in soil, thus obtaining high yield productions. The actual scenario of their inefficient use (2 million tons per year in 2014)^7^ makes it necessary to develop more efficient and less toxic methods against pests and infections, while improving crop productivity.

With this general aim, agrochemical delivery systems have been proposed as a novel class of plant protection and growth products, that promise a number of benefits in agriculture: (i) controlled release of active ingredients (AIs) in a targeted manner, avoiding contamination; (ii) improved solubility and stability of labile agrochemicals; (iii) enhanced AI retention; and (iv) improved AI absorption and uptake.^8,9^ Among novel technologies, calcium phosphate nanoparticles (NPs), mainly nanocrystalline hydroxyapatite [HA, Ca_10_(PO_4_)_6_(OH)_2_] mimicking the mineral phase of bone, have been recently proposed as a fertilizer.^10^ Aside from its high biocompatibility and biodegradability,^11,12^ HA can gradually release Ca and P (two relevant plant nutrients) in response to pH changes,^13−15^ avoiding the negative effects of soluble phosphorus fertilizers. HA NPs have demonstrated a growth effect on plants (i.e., corn, soybean, sorghum, pakchoi, pea, and rice), attributed to a slow release of phosphorus.^16,17^ Furthermore, the structure and chemical composition of HA can be manipulated toward specific functionalities (i.e., urea^18,19^ and elicitors).^20,21^

On the other hand, metal–organic frameworks (MOFs) have been recently proposed as agrochemical delivery systems.^22,23^ MOFs are an outstanding class of crystalline materials based on organic linkers (carboxylate and phosphonate) coordinated to metallic centers (atoms, chains, and clusters) resulting in one-, two-, or three-dimensional structures that are potentially porous.^24^ Derived from their “*a la carte*” structure and composition, MOFs are attractive materials as agrochemical delivery systems due to their interesting properties: (i) large specific surface areas and pore volumes, related to exceptional sorption capacities;^25^ (ii) active sites, where adsorbates can be anchored; (iii) easily functionalizable cavities, where specific host–guest interactions may take place, allowing the reversible adsorption/desorption process; (iv) the possibility of using healthy, friendly, or active constituents (cations or organic linkers); and (v) some are commercially available and can be synthesized at a large scale. Particularly, MOFs and MOF-composites have been recently reported in the controlled release of agrochemicals. On recent example is the Fe-MOF, based on urea and oxalic acid, synthesized in the laboratory and pilot scale with similar yields (27 %), which demonstrated to be efficient in the release of several plant nutrients (N, P, and Fe), promoting the rice yield.^26^ Another Fe-based material [MIL-100(Fe)] was loaded with the fungicide azoxystrobin (16.2 wt %) that exhibits good fungicidal activities against two pathogenic fungi—wheat head scab (*Fusarium graminearum*) and tomato late blight (*Phytophthora infestans*).^27^ Finally, we want to mention the agrochemical-based MOF (GR-MOF-7), build up from the herbicide glufosinate and the widely used antibacterial and fungicide Cu^2+^, which demonstrated a combined antibacterial (against *Staphylococcus aureus* and *Escherichia coli* using ≤2.5 ppm) and herbicide (against *Raphanus sativus* only with 1 mL of 0.01 M) activities.^28^

In this work, we want to go a step further in the promising role of agrochemical delivery systems in agriculture, using for the first time a composite based on HA and MOFs with the aim of combining and improving the properties of raw materials. In particular, we selected the benchmarked zeolitic-like MOF ZIF-8 [Zn(Hmim)_2_] (Hmim = 2-methylimidazole). ZIF-8 is based on Zn, one essential plant micronutrient, that shows good biocompatibility.^29,30^ Furthermore, ZIF-8 can efficiently inactivate both Gram-negative and Gram-positive bacteria by the production of reactive oxygen species under UV-sunlight generating oxidative stress in the bacteria,^31^ as previously demonstrated with other Zn-based MOFs with excellent antibacterial activity.^32^

Thus, this article reports the synthesis and complete characterization of a ZIF-8@HA composite and its evaluation as a fertilizer and antibacterial agent. This novel multitarget composite material can release in a controlled manner several micronutrients (Ca, P, and Zn), leading to a higher yield in wheat growth and improving the antibacterial activity against *Pseudomonas syringae* (*Ps*). The ZIF-8@HA composite might be considered an attractive formulation prototype for more efficient multifunctional agrochemicals and a starting point in the development of MOF@HA composites.

## 2 Methodology

### 2.1 Synthesis of ZIF-8

ZIF-8 was synthesized according to a previously reported method.^33^ In a typical procedure, 0.744 g (2.5 mmol) of Zn(NO_3_)_2_·6H_2_O was dissolved in 10 mL of deionized water and added to a solution consisting of 8.2 g of 2-methylimidazole (Hmim, 0.1 mol) in 90 mL of deionized water. The mixture was stirred at room temperature. The solution quickly became cloudy, and a suspension was obtained. After 24 h, the suspension was filtered and washed with deionized water.

All chemicals were commercially purchased and used without further modification. 2-Methylimidazole (Hmim, 99 %), Zn(NO_3_)_2_·6H_2_O (98 %), CaCl_2_ (99.99 %), Na_3_(C_6_H_5_O_7_) (>99.9 %), Na_2_HPO_4_ (>99.0 %), and Na_2_CO_3_ (>99.5 %) were obtained from Acros Organics.

### 2.2 Synthesis of Biomimetic HA NPs

HA NPs were synthesized following a batch precipitation protocol consisting in mixing two solutions of equal volume: (A) CaCl_2_ (0.2 M) and Na_3_(C_6_H_5_O_7_) (0.2 M), and (B) Na_2_HPO_4_ (0.12 M) and Na_2_CO_3_ (0.1 M) at 60 °C for 24 h.^19,34,35^ Then, the precipitates were collected and repeatedly washed with ultrapure water by centrifugation (5000 rpm, 15 min, and 18 °C).

### 2.3 Synthesis of ZIF-8@HA

After selecting 1000:1 as the optimal ZIF-8/HA ratio to obtain the ZIF-8@HA composite (Section S2, Supporting Information, SI), the synthesis was scaled up 10 times. A suspension containing 40.7 mg (0.08 mmol) of HA in 10 mL of deionized water was first sonicated in an ultrasonic bath for 10 min. Then, a solution of 740 mg (2.5 mmol) of Zn(NO_3_)_2_·6H_2_O in 10 mL of deionized water was added, and the mixture was stirred for 5 min. Finally, an aqueous solution of 8.2 g (100 mmol) of Hmim in 90 mL of deionized water was added, and the resulting mixture was stirred for 24 h. The obtained solid was filtered, washed twice with deionized water, and dried at room temperature (94 % yield, *ca.* 0.6 g). All of the obtained materials were further characterized as described in Sections S1–S2, Supporting Information.

### 2.4 Stability in Aqueous and King’s B Media

First, the stability of the obtained composite was determined in deionized water by measuring the release of the Hmim ligand by UV–vis spectroscopy. Water was selected as it is normally used to apply agrochemicals in fields as suspensions or solutions. 20 mg of ZIF-8@HA was suspended in 4 mL of water and incubated under bidimensional stirring at room temperature (20–25 °C). At different incubation times (0, 10, 20, 30, and 45 min and 1, 2, 3, 4, 8, 24, 48, 72, 96, 144, and 168 h), an aliquot of 2 mL was analyzed, and the same volume of water was added to the suspension in order to keep sink conditions. The kinetic study was carried out in triplicate (*n* = 3). The structural integrity of ZIF-8 was also analyzed by X-ray powder diffraction (XRPD) at different times (24, 72, and 168 h) (Supporting Information, Figure S6).

Then, the stability of the ZIF-8@HA composite was also studied in King’s B (KB) medium (pH = 4.5), which is employed in the antibacterial tests. 2 mg of ZIF-8@HA was suspended in 2 mL of KB medium under bidimensional stirring at 26 °C. At different incubation times (10, 20, 30, and 45 min and 1, 2, 3, 4, 8, and 24 h), an aliquot of 1 mL was analyzed by inductively coupled plasma mass spectrometry to determine the potential release of Zn^2+^ and the same volume of water was added to the suspension to keep sink conditions. The same experiment was performed with ZIF-8 and a physical mixture of ZIF-8 and HA as controls. This study was performed in triplicate. The statistical analyses were performed using the one-way ANOVA with a *p*-value < 0.05 in both aqueous and KB media (Supporting Information, Figure S8).

### 2.5 Nutritional Effect in Wheat

Wheat seeds (*Triticum aestivum*) were supplied by Agrointec (Almería, Spain). Before use, seeds were stored in a dry dark place at room temperature. Initially, wheat seeds were germinated in wet paper, under dark conditions at 25 °C, obtaining a high rate of germination (*ca.* 90 %) after 2 days of incubation (root seed length *ca*. 0.5 mm).

First, the optimal concentration of ZIF-8@HA was determined. Various parameters (shoot and root length) were measured to evaluate the fertilizer effect of the composite. Seedlings were divided into 4 different groups (*ca.* 30 seedlings per group) and placed in glass vials and 1 mL of ZIF-8@HA suspension at different concentrations (0, 7.3, 73, and 730 mg·L^–1^) was added. Plants were kept at ambient conditions of humidity, in the dark, and at 25 °C for 11 days. An increment in the root length was observed when using 7.3 mg·L^–1^ of ZIF-8@HA. However, higher concentrations (73 and 730 mg·L^–1^) inhibit root growth (Figure S7), probably related with higher doses of Zn^2+^ as previously reported.^36^ Thus, 7.3 mg·L^–1^ was considered the optimal concentration for the composite wheat seeds’ development.

Once the optimal ZIF-8@HA concentration was determined, the efficiency of ZIF-8@HA in seed development was further studied. Again, seedlings were divided into six different groups: (i) water (control), (ii) ZIF-8 (7 mg·mL^–1^), (iii) HA (0.3 mg·mL^–1^), (iv) ZnSO_4_ (4.9 mg·mL^–1^), (v) a physical mixture of HA (0.3 mg·mL^–1^) and ZIF-8 (7 mg·mL^–1^), and (vi) ZIF-8@HA (7.3 mg·mL^–1^). Note that the studied amount of material was adjusted to its corresponding concentration in ZIF-8@HA. Between 40 and 60 seedlings per group were placed individually in glass vials, and 1 mL of the corresponding suspension or aqueous solution was added. Plants were kept under ambient conditions of humidity, in the dark at 25 °C. All measurements were represented as average and standard deviation and analyzed statistically using a two-way ANOVA test to determine the significance between the average of the different treatments, with a statistical significance of 0.001 and 0.01.

### 2.6 Antibacterial Experiments

*Ps* (CECT 126) was purchased at the Colección Española de Cultivos Tipo (CECT). The pathogenic bacteria were incubated in tryptic soy broth (TSB, no. 2, Sigma-Aldrich) at 26 °C overnight, following the supplier recommendations. A stock bacterial suspension was prepared by introducing viable*Ps*into KB medium to attain a final optical density (O.D.) of around 0.1, according to a previous study.^37^ The following treatments were assessed: (i) bacterial control (*control*), (ii) *Ps* in the presence of ZnSO_4_ (Zn^2+^), (iii) *Ps* in the presence of ZIF-8@HA (ZIF-8@HA), and (iv) *Ps* in the presence of ZIF-8 (ZIF-8), all of them at 50 ppm of Zn. Additional details regarding the experimental conditions can be found in Table S1. The assay was carried out at 30 °C using 96-well plates, and the evolution of the absorbance with time was measured in a NanoQuant microplate reader (Thermo Fisher Scientific). For each condition, blank curves were generated by substituting 20 μL of the stock bacterial suspension in KB with 20 μL of KB medium. The absorbance value of the blanks was subtracted from the absorbance of the bacterial growth curves (raw data in Figure S9) and then normalized as growth rate (%) with respect to the absorbance value of the control. Triplicate experiments were performed for each condition.

## 3 Results and Discussion

### 3.1 Synthesis and Characterization of ZIF-8@HA

**Figure 1:**
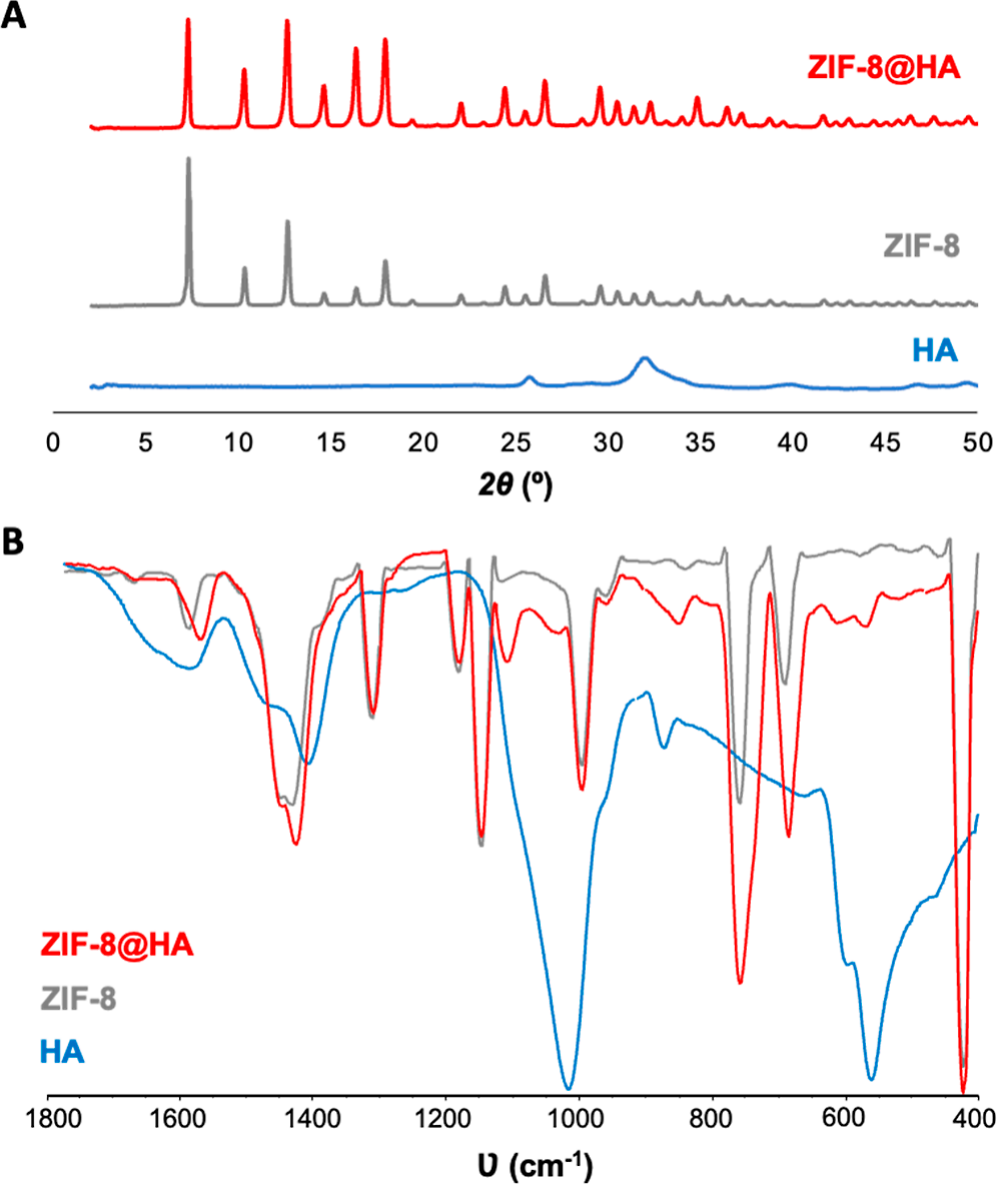
(A) XRPD patterns and (B) Fourier-transform infrared (FTIR) spectra of ZIF-8@HA composite (red) and raw materials (ZIF-8, gray; HA, blue).

The synthesis of ZIF-8 was carried out at room temperature in aqueous media and in the presence of HA NPs (previously grown by the method described in Section 2.2), avoiding the use of hazardous solvents and under mild conditions (neutral pH, room temperature, and atmospheric pressure). The design of the best synthetic strategy was optimized by evaluating increasing ZIF-8/HA ratios, as described in Section S2, Supporting Information. Following this strategy, the HA NPs remained on the surface, decorating the surface of ZIF-8. The XRPD pattern of the optimized ZIF-8@HA composite (Figure 1A) displays the characteristic reflections of the ZIF-8 structure, indicating the successful formation of the MOF in aqueous solution containing HA NPs. The ZIF-8@HA composite FTIR spectrum (Figure 1B) shows the main vibrational modes of ZIF-8 such as the Zn–N bond at 420 cm^–1^, C=C at 758 and 693–684 cm^–1^, and C–N at 1450–1300 cm^–1^, along with the characteristic triply degenerate bending mode of phosphate groups (ν_4PO4_) at *ca.* 602 and 561 cm^–1^ of HA.^38^

**Figure 2:**
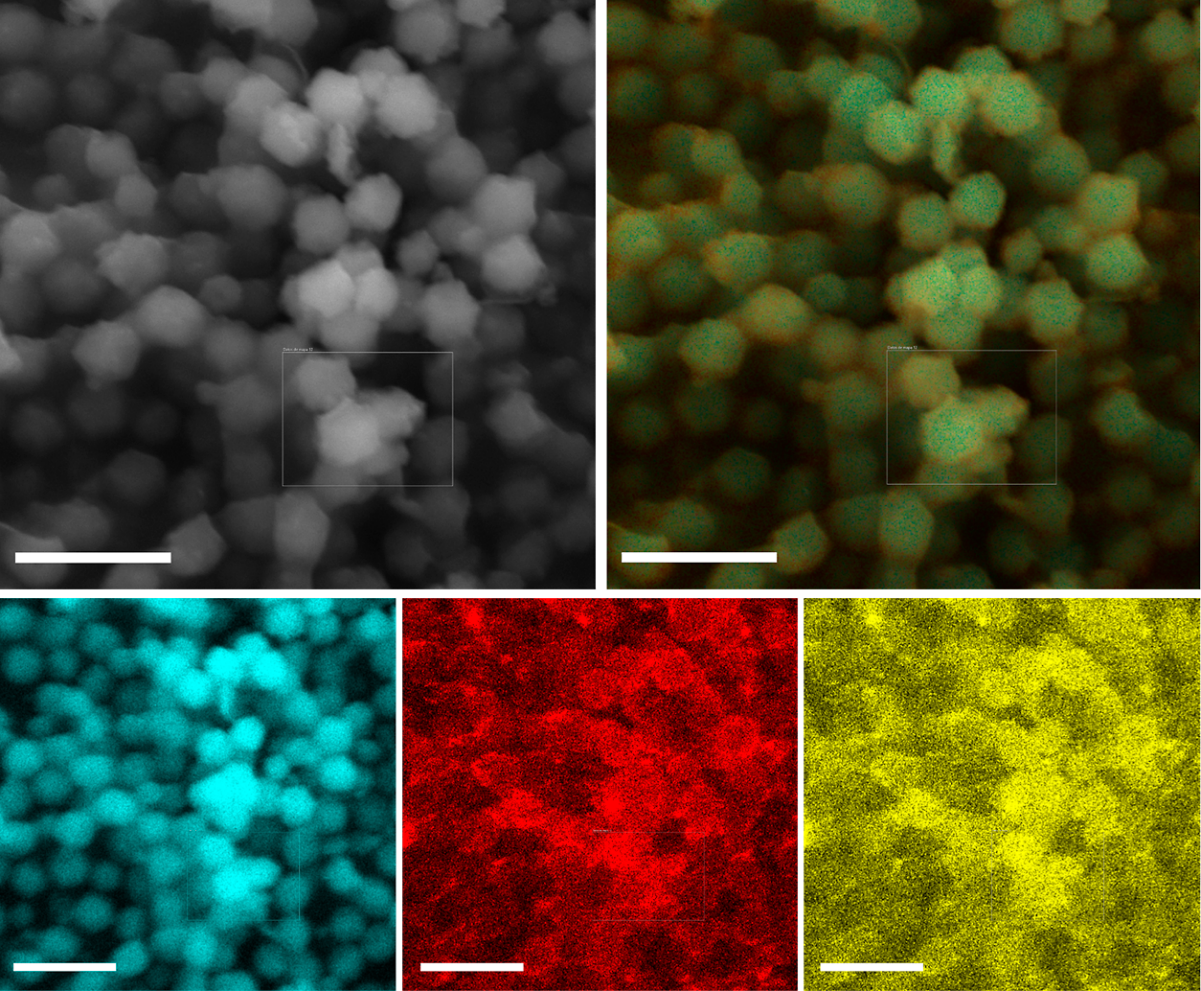
SEM image and energy-dispersive X-ray spectroscopy (EDS) elemental maps of ZIF-8@HA showing the spatial distribution of Zn (cyan), Ca (red), and P (yellow). Scale bars correspond to 2.5 μm.

**Figure 3:**
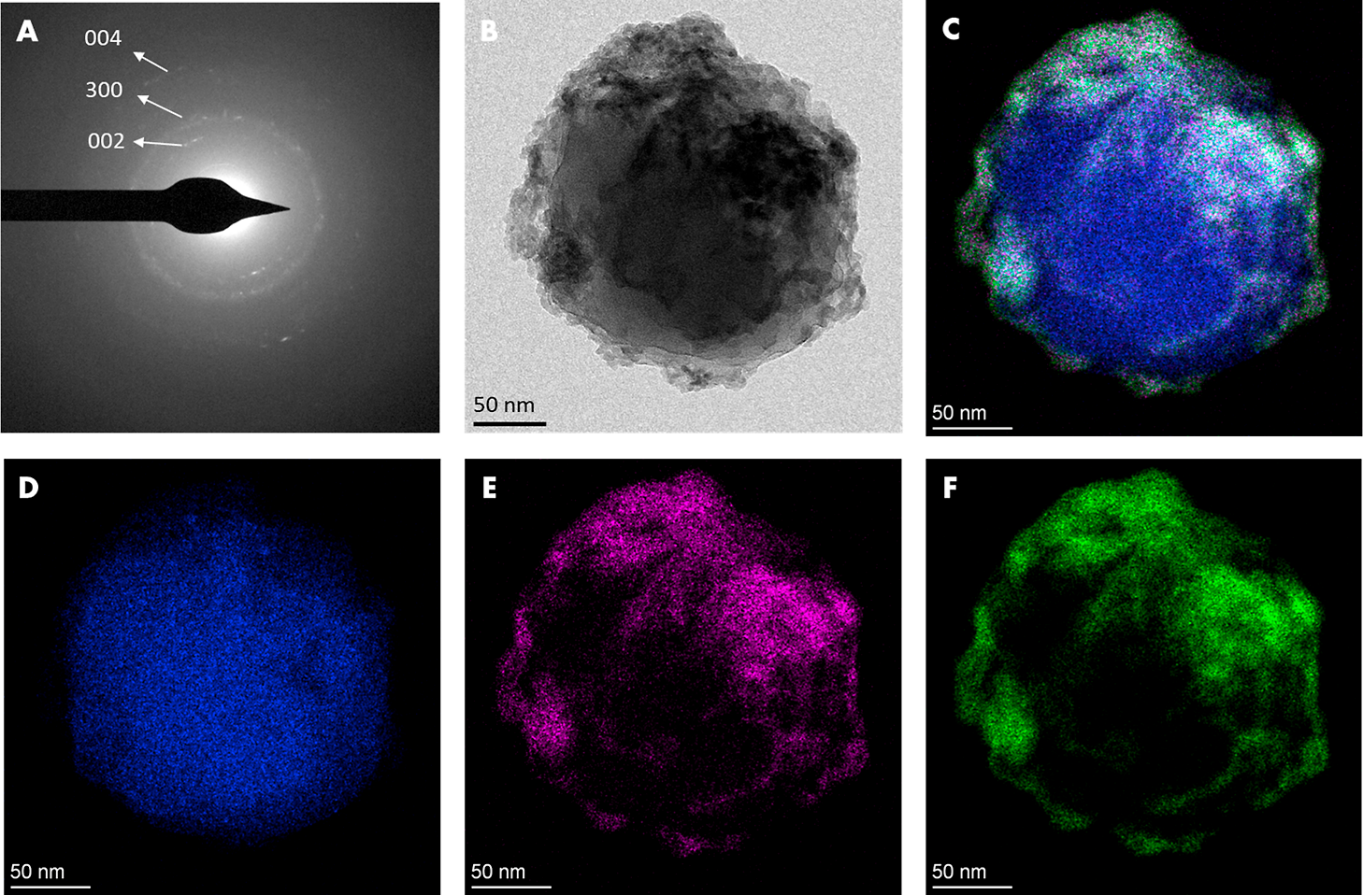
TEM analysis of ZIF-8@HA. (A) Selected area electron diffraction (SAED). (B) TEM image. (C) EDS maps merging Zn, P, and Ca and of (D) Zn, (E) P, and (F) Ca.

Scanning electron microscopy (SEM) images of the ZIF-8@HA composite show spherical particles like those of naked ZIF-8 (Figures 2 and S3). The mean diameter of ZIF-8@HA (1061 ± 149 nm) is larger than ZIF-8 particles (662 ± 135 nm), which suggest the presence of HA aggregates on the external surface of ZIF-8. The EDS elemental maps (Figure 2) confirmed the presence of calcium (red) and phosphorus (yellow) evenly distributed across the ZIF-8 particle along Zn (cyan), indicating the formation of a hybrid structure in which the HA NPs are surrounding ZIF-8. Transmission electron microscopy (TEM) analysis of an isolated ZIF-8@HA NP (Figure 3) also showed a core–shell structure, where HA is covering ZIF-8. The nature of the coating was confirmed by SAED of the composite (Figure 3A) which displays the characteristic reflections of HA (002, 300, and 004, ASTM Card file no. 9-432).

Aside from microscopy studies, the analysis of the surface charge is an extremely valuable characterization to describe the effectiveness of the MOF-HA formation since a simple modification of the particle surface may provoke substantial differences in the charge of the surface. Electrophoretic light scattering analysis in water of ZIF-8@HA revealed a ζ-potential of −27.6 ± 0.7 mV, whereas control ZIF-8 particles have a positive surface charge (ζ-potential = 18.7 ± 0.8 mV). The radical change from positive to negative ζ-potential values confirmed that negatively charged HA NPs (ζ-potential = −30.7 ± 2.0 mV) covered the ZIF-8 surface. All these results suggest that ZIF-8 particles of around 500–800 nm were first grown and then covered by HA NPs (nanoplatelets of around 100 nm), through a process probably directed by electrostatic interactions, resulting in hybrid materials with a core@shell structure.

The chemical composition of dehydrated samples (95 °C overnight) was evaluated by elemental analysis (EA), inductively coupled plasma optical emission spectroscopy (ICP-OES), and thermogravimetric analysis (TGA). The proposed chemical formula of ZIF-8 Zn(C_4_H_5_N_2_)_2_ is in agreement with the one previously published.^33^ Considering the ICP-OES values (Table 1) obtained for Zn (59.73 %), Ca (5.36 %), and P (3.35 %), one can set a Zn/Ca molar ratio of 11.15:1. Furthermore, considering the composition of ZIF-8 (one Zn per mol) and HA (5 mol of Ca per mol), one can set a molecular ratio ZIF-8/HA of 55.7 to 1, leading to the chemical formula (ZIF-8)_55.7_(HA) or [Zn(C_4_H_5_N_2_)_2_]_55.7_[Ca_5_(PO_4_)_3_(OH)]. These values agree with those obtained with other techniques, like EA (Table 1) or TGA (Figure S4). Thus, the proposed formula with combined composition for ZIF-8@HA is [Zn(C_4_H_5_N_2_)_2_]_55.7_[Ca_5_(PO_4_)_3_(OH)] (MW: 13178.62 g·mol^–1^). C(40.61 %), H(4.27 %), and N(23.68 %); found: C(42.64 %), H(6.72 %), and N(25.30 %).

**Table 1:** Chemical Composition by ICP-OES (Molar Ratio) and EA (wt %) of ZIF-8@HA

	ICP-OES (molar ratio)			EA (wt %)		
	Ca	P	Zn	C	N	H
ZIF-8@HA	5.36	3.35	59.73	42.64	25.30	6.72

### 3.2 Stability in an Aqueous Medium

**Figure 4:**
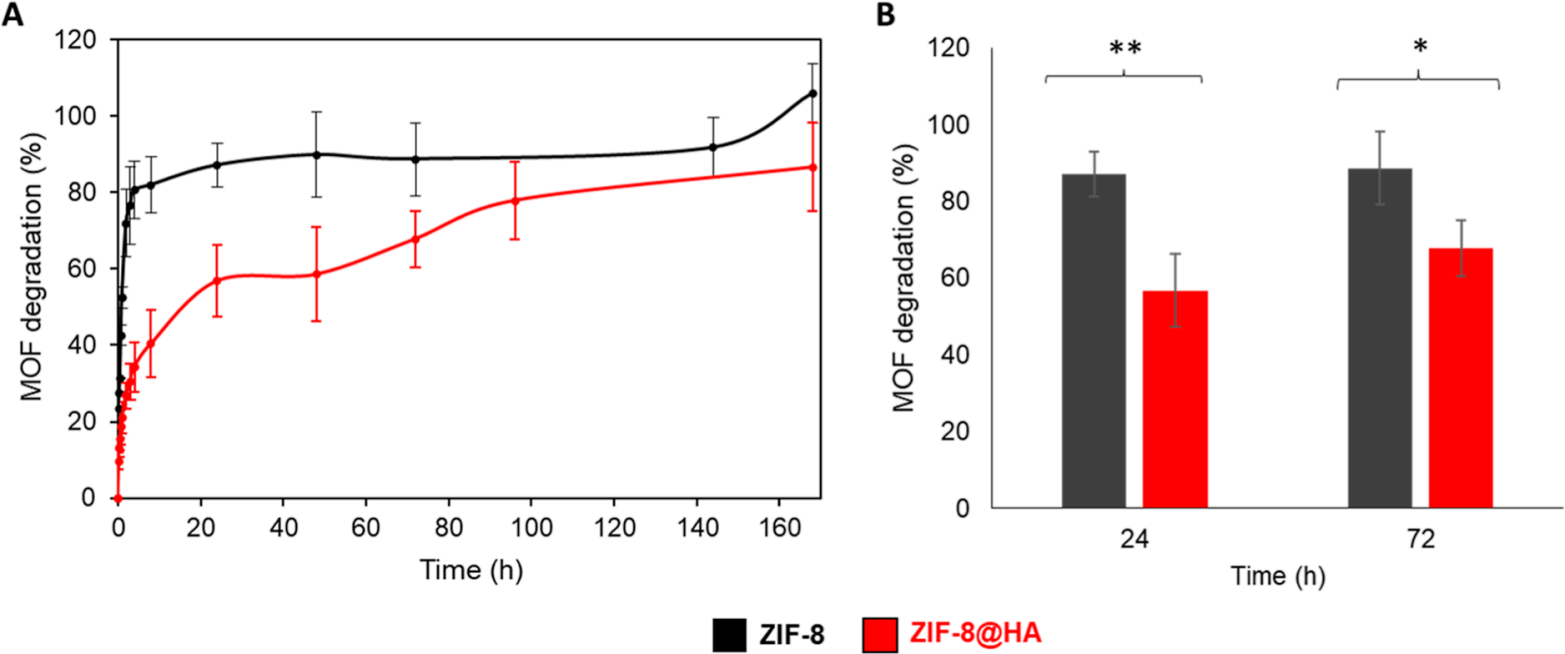
(A) ZIF-8 and ZIF-8@HA stability in aqueous media monitored by UV–vis spectroscopy up to 168 h at room temperature. (B) Statistical analysis of MOF degradation after 24 and 72 h using the one-way ANOVA where *p-value < 0.05 and ** *p*-value < 0.01. The analysis was performed in triplicate and all measurements are represented as average and standard deviation.

Agrochemicals are normally sprayed as an aqueous solution or suspension in the field in order to reach different parts of plants or the ground. The chemical stability of ZIF-8@HA in water was investigated by UV–vis spectroscopy by means of the linker (Hmim) release for 7 days (168 h, Figure 4). Interestingly, the stabilization effect of the HA core–shell around ZIF-8 NPs was confirmed, as the degradation of the MOF is delayed from 4 h for pristine ZIF-8 to 168 h for the ZIF-8@HA composite. Furthermore, the structural stability of the MOF inside the composite was monitored by XRPD. After 7 days, ZIF-8 keeps its crystalline structure, although additional diffraction signals are observed (Supporting Information, Figure S6).

### 3.3 Evaluation of the Nutritional Effect of ZIF-8@HA in Wheat

**Figure 5:**
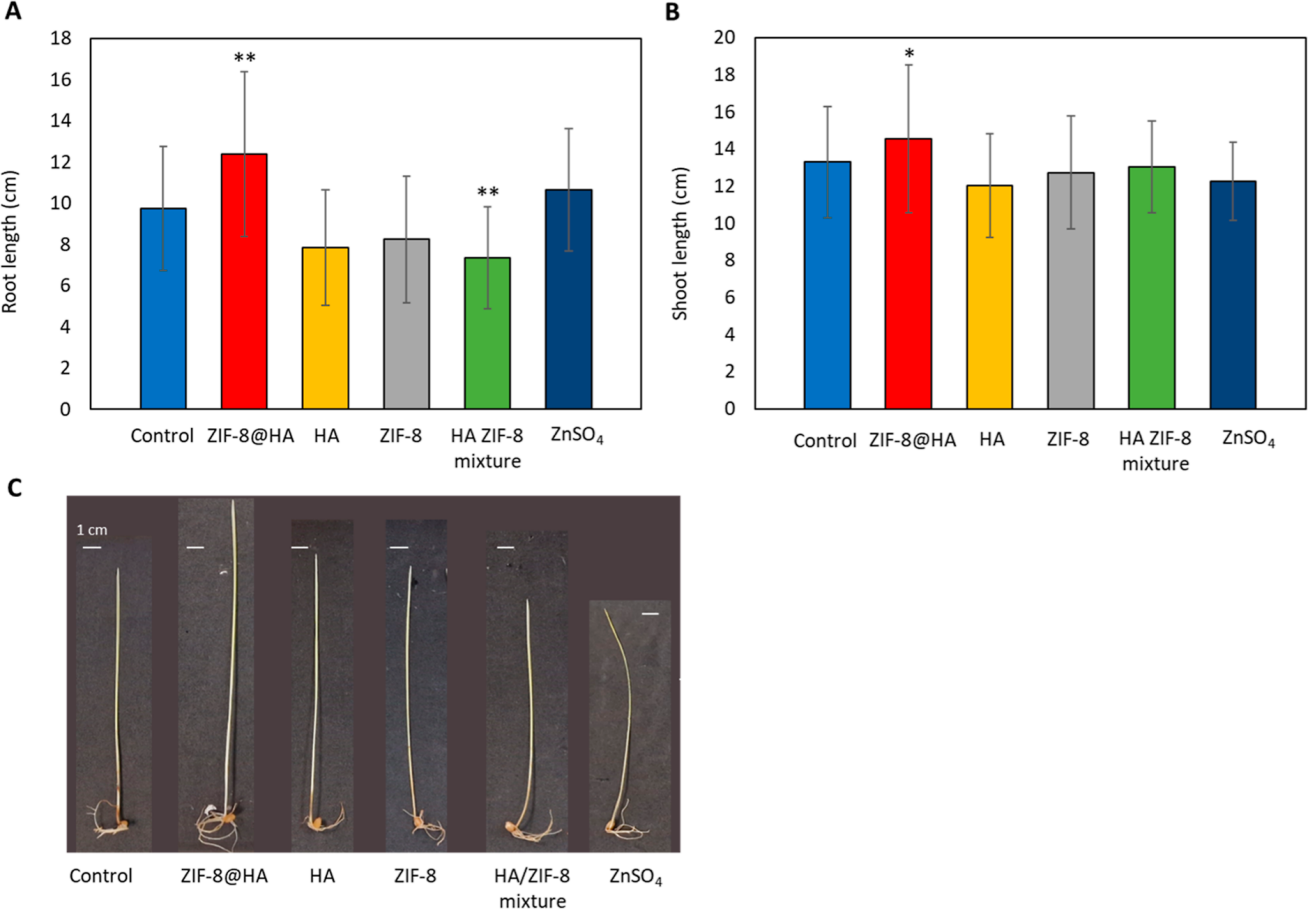
Root length (A), shoot length (B), and a representative image of a wheat seed (C) after treatment with water (control), ZIF-8@HA composite, HA, ZIF-8, a physical mixture of both HA and ZIF-8, and ZnSO_4_ after 11 days of growth. Each treatment was assessed with *ca.* 40–60 samples, where the average and standard deviation are represented. The corresponding statistical analysis was performed for each treatment with respect to control using the one-way ANOVA test where *p < 0.01 and **p < 0.001.

The wheat (*T. aestivum*) seed growth assay was used to evaluate the effectiveness of ZIF-8@HA as a nutrient. First, the active ZIF-8@HA concentration (7.3 mg·L^–1^) was determined by submerging wheat seeds in suspensions with different ZIF-8@HA concentrations (see Section 2.5). Various parameters were measured to evaluate the fertilizer effect: shoot length and root length. After 11 days, the growth of wheat seeds in the presence of ZIF-8@HA (7.3 mg·L^–1^) resulted in an increment in both root and shoot length (27.1 and 9.4 %, Figure 5A and B, respectively) when compared with the control group (without treatment). The rest of the treatments (HA, ZIF-8, ZnSO_4_, and HA + ZIF-8 mixture) did not show significant differences in the root and shoot growth with respect to the control. Importantly, the ZIF-8@HA composite showed an improved fertilizer effect than the physical mixture of HA and ZIF-8. The significant increase in the root and shoot length provided by the ZIF-8@HA treatment can be associated with the slower Zn release compared to ZIF-8 or a physical mixture of ZIF-8 and HA (Figure 4). In this regard, Zn is an essential micronutrient playing a key role in several physiological processes of plants such as the biosynthesis of protein, enzymes, and chlorophylls or phosphate and carbohydrate metabolism.^39,40^ This enhanced plant growth effect for Zn nanoformulations compared to conventional soluble Zn fertilizers (ZnSO_4_) is in agreement with previous reports on wheat, as well as other crops such as coffee plants, peppers, or tomatoes.^40^

### 3.4 Antibacterial Effect

**Figure 6:**
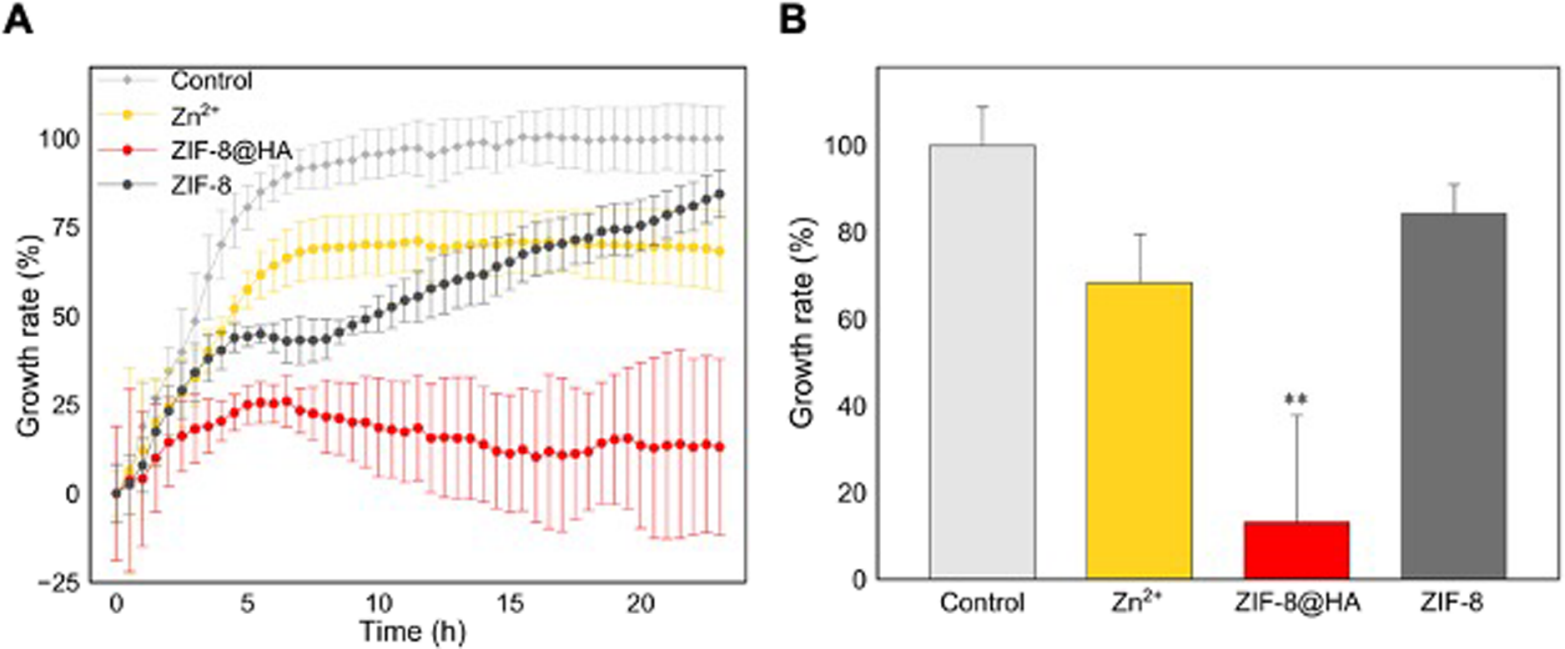
(A) Growth rate of *Ps* in the presence of zinc sulfate (Zn^2+^), ZIF-8@HA composite, and pristine ZIF-8 and (B) maximum growth reached for each treatment after 23 h. Treatments were assessed in triplicates and the corresponding statistical analysis was performed using the one-way ANOVA test and Bonferroni’s posthoc test, where **p-value <0.01.

Zn^2+^ ions are able to inhibit the growth of relevant vegetal pathogens such as *Ps*,^34,41,42^ the principal bacteria responsible for wheat crop decline, with high incidence in wheat crops (5–50 % of losses).^43^ Initially, the chemical stability of ZIF-8@HA was evaluated in KB media, which was used to grow *Ps* (Section 2.6, Figure S8). At 24 h, significant differences (*p*-value < 0.05) are found between ZIF-8@HA, ZIF-8, and their physical mixture, with 67.9 ± 4.3, 88.5 ± 5.2, and 86.0 ± 2.7 % of the total Zn^2+^ released, respectively. ZIF-8 and a physical mixture of ZIF-8 and HA showed a faster Zn release than the composite ZIF-8@HA, which is in agreement with the higher chemical stability of the composite in water (Figure 4) due to the stabilization effect of HA covering. Then, the antibacterial activity against *Ps* growth was monitored through the O.D. at 630 nm (O.D.)^37^ (see Section S5 for further details). Figure 6A displays the growth rate of *Ps* in the presence of ZIF-8@HA, ZIF-8, and Zn^2+^, all of them at 50 ppm of Zn^2+^. While ZIF-8@HA induced a prominent inhibition of bacterial proliferation (>80 % of growth inhibition), this effect was less notorious or even not observed with Zn^2+^ and ZIF-8. In fact, ZIF-8@HA exhibited statistically significant growth inhibition of *Ps* after 23 h, whereas ZIF-8 or Zn^2+^ did not (Figure 6B). The combinatorial inhibition effect of ZIF-8 and HA NPs is ruled out since calcium phosphate NPs enhance *Ps* proliferation, as previously reported.^37^ Thus, the highest inhibition activity of ZIF-8@HA can be attributed to the stabilization effect of HA on its degradation profile, in agreement with the stability test (Figure S8). Thus, ZIF-8@HA acts as a metal reservoir, making it more effective in the inhibition of the pathogen growth, as previously reported in other Zn-based materials.^32^ These results demonstrate the potential of MOF@HA composites to develop multifunctional agrochemicals, improving the fertilizer and antibacterial effect of the individual components, HA and MOF, respectively.

### 3.5 Scale-Up and Life Cycle Assessment

The production of the materials at a large scale and the corresponding life cycle assessment (LCA) need to be explored for their widespread application. The individual components HA and ZIF-8 are already manufactured and traded by the industry for cosmetics or medicine.^44−46^ Moreover, pilot-scale studies for kg of ZIF-8 and calcium phosphate NPs (i.e., amorphous calcium phosphate, the precursor of HA) have been already carried out, demonstrating the feasibility of its scaling. Using analytical-grade reagents, the cost of the ZIF-8@HA composite is around 2.77 €/g, 90 % relying on the 2-methylimidazole reagent. This price can be significantly reduced by the use of low-cost technical-grade reagents, following an approach similar to that previously carried out to reduce by 35 times the price of calcium phosphate NPs,^37^ or by recycling of the mother liquors containing unreacted 2-methylimidazole.^49^ Considering the optimum concentration of ZIF-8@HA (7.3 mg·L^–1^), the treatment cost per seed will be 0.00067 €. Despite the fact that the cost of the marketed zinc suppliers (e.g., ZnSO_4_) is much lower than ZIF-8@HA, its lower efficiency due to fertilizer losses associated with the high solubility and low retention of the conventional fertilizer, and the consequent environmental costs, should be considered.

LCA is defined as an environmental management tool for the holistic, systematic, and multidisciplinary qualification and quantification of the environmental impacts and damages of a product or process throughout their entire lifecycle.^47^ The LCA methodology, according to International Standards 14040 and 14044, comprises four iterative steps: (i) goal and scope definition, (ii) inventory analysis, (iii) impact assessment, and (iv) interpretation.^48,49^ Up to the present, there have been several studies focused on the evaluation of ZIF-8 on different applications such as gas separation, catalysis, water splitting, and adsorption.^47^ LCA of HA NPs for sunscreen has also been evaluated.^50^ Nonetheless, the LCA of ZIF-8, HA, or composite materials such as ZIF-8@HA for agricultural practices has never been evaluated. In this work, the synthesis of ZIF-8 and HA was in an aqueous environment and under mild conditions (neutral pH, room temperature, and atmospheric pressure), avoiding the use of hazardous solvents or extreme reaction conditions. Respecting the production process, a previous study indicated that aqueous synthesis of ZIF-8 is among the four synthesis methods with the lowest environmental impact.^47^ Since the reagent with the largest environmental burden is 2-methylimidazole due to its health hazard (i.e., irritation of skin, eyes, and respiratory system), the recycling of the mother liquors^48^ can reduce the environmental impact of the production process. Regarding the environmental impact of ZIF-8@HA application in agriculture, both HA and ZIF-8 are considered nontoxic and biocompatible.^12^ The ZIF-8@HA showed high stability in an aqueous environment, providing a gradual release of its constituents (Ca, P, and Zn), increasing the efficiency, and reducing the environmental impact of the usage of highly soluble conventional fertilizers. Moreover, the plant protection effect of ZIF-8@HA against *Ps* can reduce the usage of environmentally harmful conventional pesticides in agriculture, as previously described.^40^ Overall, ZIF-8@HA presents more efficient and sustainable strategies toward plant nutrition and protection due to its dual functionality, chemical stability, and biocompatibility.

## 4 Conclusions

In this study, we developed a multifunctional slow-release agrochemical via a simple and efficient method combining ZIF-8, a porous material containing Zn with antibacterial potential, with HA NPs, a promising phosphate nanofertilizer. XRPD, SEM, TEM, and surface charge analysis of the optimized ZIF-8@HA composite confirmed the external surface modification of ZIF-8 with HA in a core–shell manner, without affecting the crystalline structure of the MOF. Moreover, the HA coating improved the chemical and structural stability of the MOF in water and KB media, leading to a controlled release of its components. Wheat seed growth assays revealed an improved fertilizer potential of the composite material compared with the individual components or their sum. The ZIF-8@HA composite also enhanced the antibacterial properties against *Ps*, the principal bacteria responsible for wheat crop decline. All of these features make ZIF-8@HA NPs a very promising candidate in agriculture. Further experimental works are being done to introduce a third AI in the pores of the material to exploit further this well-known property of the ZIF-8 MOF.

## Supporting Information Available

Instruments used in the physicochemical characterization of compounds; experimental details of the synthesis and characterization of ZIF-8@HA including XRPD patterns of ZIF-8@HA using different ZIF-8:HA ratios, TEM images of the solid obtained when using 100:1 and 1000:1 ZIF-8:HA ratios, SEM images of ZIF-8 and ZIF-8@HA, TGA of HA, ZIF-8, and ZIF-8@HA composite, and XRPD patterns of the residues obtained after TGA analysis of ZIF-8, HA, and ZIF-8@HA composite; ZIF-8@HA stability study; nutritional effect in wheat; and antibacterial experiments (PDF)
