# Supplementary material for: ZIF-8@Hydroxyapatite Composite as a High Potential Material for Prolonged Delivery of Agrochemicals
Source: ACS Appl Mater Interfaces. 2024 May 27;16(22):29305–13. doi: 10.1021/acsami.4c06016 (PMC11163398; doi:10.1021/acsami.4c06016)
Supplement: Supplementary file 1 — am4c06016_si_001.pdf [file am4c06016_si_001.pdf]

Supporting Information

ZIF-8@Hydroxyapatite composite as high potential material for prolonged delivery of agrochemicals

Samuel Morales-Cámara, Belén Parra-Torrejón, Antonio Rodríguez-Diéguez, José M. Delgado-López, Gloria B. Ramírez-Rodríguez and Sara Rojas**

Department of Inorganic Chemistry, University of Granada, Av. Fuente Nueva, s/n, 18071, Granada, Spain

Corresponding Authors: srojas@ugr.es, gloria@ugr.es

Table of contents

S1. Physicochemical characterization **2**

S2. Synthesis and characterization of ZIF-8@HA composite **4**

S3. ZIF-8@HA stability in aqueous media **8**

S4. Nutritional effect in wheat **9**

S5. Antibacterial experiments **10**

S1. Physicochemical characterization

Elemental analyses (EA) were carried out on a Thermo Scientific analyzer model Flash 2000 from Centre for Scientific Instrumentation of the University of Granada (CIC-UGR). The Fourier transform infrared (FTIR) spectra, measured on powdered samples in an attenuated total reflectance (ATR) mode, were recorded on a BRUKER TENSOR 27 FT-IR and OPUS data collection program. X-ray powder diffraction (XRPD) patterns of all samples were collected in a BRUKER D8 ADVANCE equipment (CIC-UGR), where the routine PXRD conditions were from 3 to 35º (2θ) using a step size of 0.013º and 39525s *per* step in continuous mode with knife and Soller slits of 0.04 rad. Thermogravimetric analyses (TGA) were carried out in a thermogravimetric analyzer mod. TGA/DSC1 (CIC-UGR, Mettler Toledo, Columbus, OH, USA) with a general heating profile from 30 to 900 ºC with a heating rate of 10 ºC min^{-1} under air using a flux of 100 $mL \cdot min^{-1}$. Transmission Electron Microscopy (TEM) was carried out using a TEM HAADF TALOS F200X (Thermo Fisher Scientific, Waltham, MA, USA) coupled with Energy Dispersive X-ray spectroscopy (EDX) from CIC-UGR. UV-visible spectra were registered using the Cary 60 UV-Vis spectrophotometer (Agilent Technology, Santa Clara, CA, USA). ZIF-8@HA was also imaged with a field emission scanning electron microscopy (FESEM, GEMINI CARL ZEISS) from CIC-UGR. The compositional analysis (Ca, P and Zn) was acquired with an Oxford energy dispersive spectroscopy (EDS) detector. To this aim, the samples were mounted on aluminum stubs using a carbon tape and sputtered with a thin carbon film (BAL-TEC MED-020, CIC-UGR). DLS and ζ-potential were performed at Litesizer 500 (Anton Paar, Graz, Austria), using quartz cuvettes and OMEGA cuvettes (ref. 225288), respectively. Inductive coupled plasma mass spectroscopy (ICP-MS) was performed in a spectrometer NexION (Perkin-Elmer,

Waltham, MA, USA) at Servicios Centrales de Apoyo a la investigación (SCAI), University of Málaga.

S2. Synthesis and characterization of ZIF-8@HA composite

During the preparation of ZIF-8@HA, different ZIF-8:HA ratios (10:1, 100:1, and 1000:1) were studied. A suspension containing 40.70, 4.07, or 0.407 mg (0.8, 0.08, or 0.008 mmol for ZIF-8:HA ratio 10:1, 100:1 or 1000:1, respectively) of HA in 1 mL of deionized water was first sonicated in an ultrasonic bath during 10 min. Then, a solution of 74 mg (0.25 mmol) of $Zn(NO_3)_2{\cdot}6H_2O$ in 1 mL of deionized water was added to the first suspension, and the mixture was stirred for 5 min. Finally, a solution of 820 mg (10 mmol) of Hmim in 9 mL of deionized water was added, and the resulting mixture was stirred for 24 h. The obtained white solid was filtered, washed twice with deionized water and dried. XRPD studies of the obtained solids demonstrated the successful formation of ZIF-8 structure when using ZIF-8:HA ratio of 100:1 and 1000:1. However, in the ZIF-8:HA ratio 10:1 synthesis the ZIF-8 structure is not obtained. (**Figure S1**).

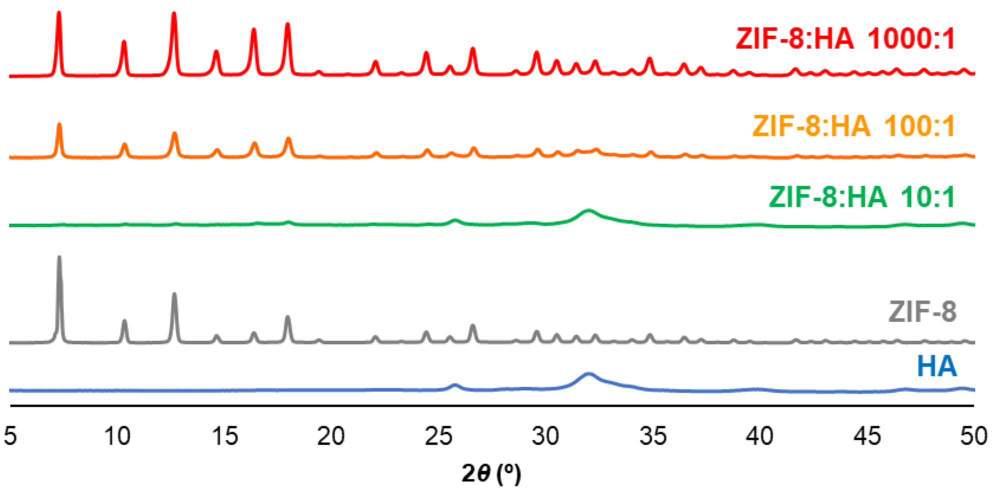

Figure S1. XRPD patterns of ZIF-8@HA using different ZIF-8:HA ratios: 10:1, 100:1 and 1000:1, and pristine ZIF-8 and HA.

TEM images of the obtained solids were recorded. When using a ZIF-8:HA ratio 100:1, the co-precipitation of HA and ZIF-8 nanoparticles (NPs) aggregates are observed. Therefore, this ratio was discarded. On the other hand, when using the 1000:1 ZIF-8:HA ratio, ZIF-8 is englobed by HA NPs (**Figure S2**).

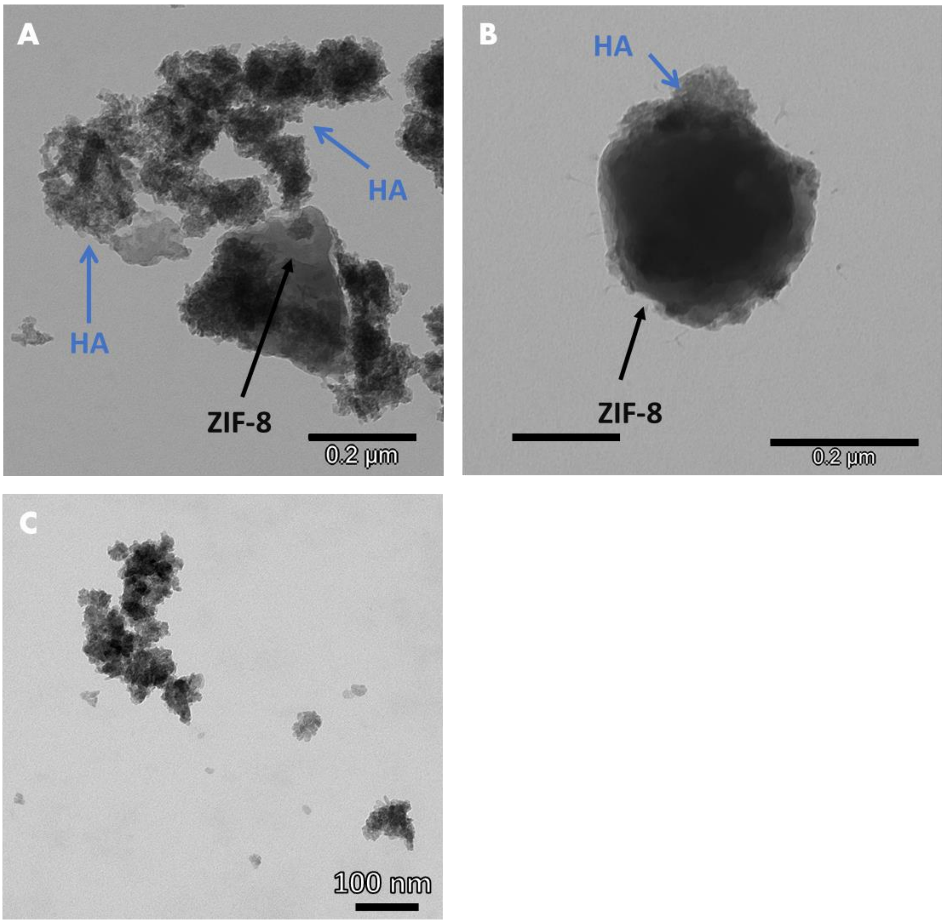

Figure S2. TEM images of the solid obtained when using (A) 100:1 and (B) 1000:1 ZIF-8:HA ratio. The microscopical analysis confirms the successful formation of a composite material with the synthesis 1000:1. (C) TEM image of HA nanoparticles has been added for comparison.

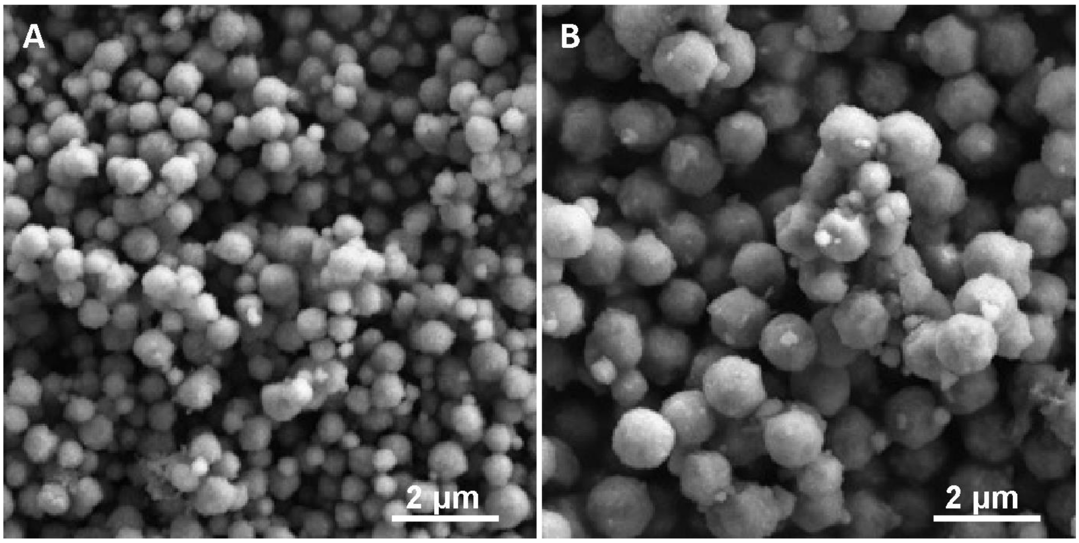

Figure S3. SEM image of (A) ZIF-8, and (B) ZIF-8@HA.

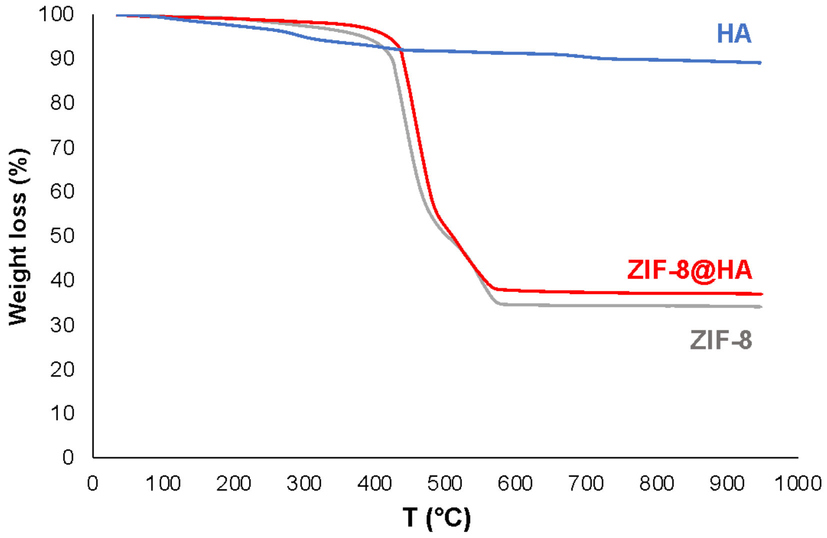

Figure S4. TGA of HA (blue), ZIF-8 (grey), and ZIF-8@HA composite (red). After the thermal treatment of ZIF-8@HA, the obtained residue was identified as dried crystalline HA and ZnO. Residue after thermal treatment: $(ZnO)_{55.7}(Ca_5(PO_4)_3)$; calculated: 37.99%; found: 37.18%.

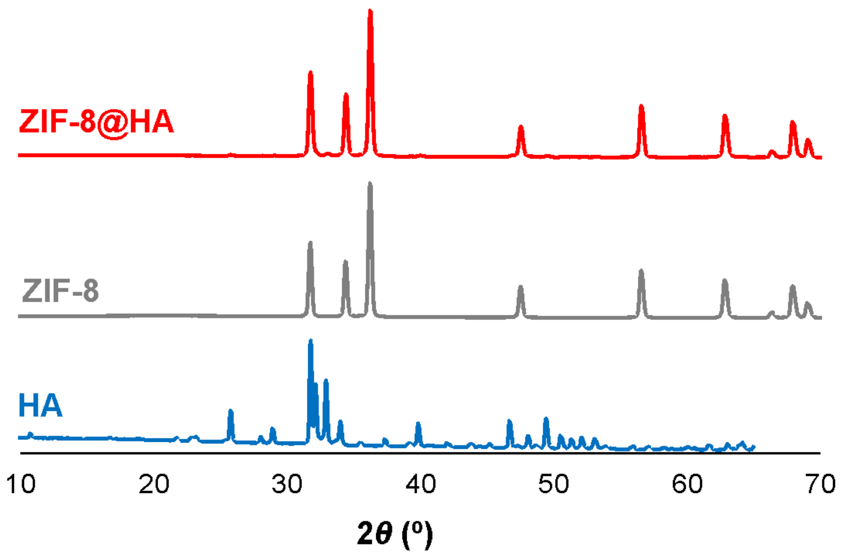

Figure S5. The XRPD patterns of the residues obtained after the TGA analysis of ZIF-8, HA, and ZIF-8@HA composite confirm the presence of HA and ZnO after ZIF-8@HA thermal degradation.

S3. ZIF-8@HA stability in aqueous media

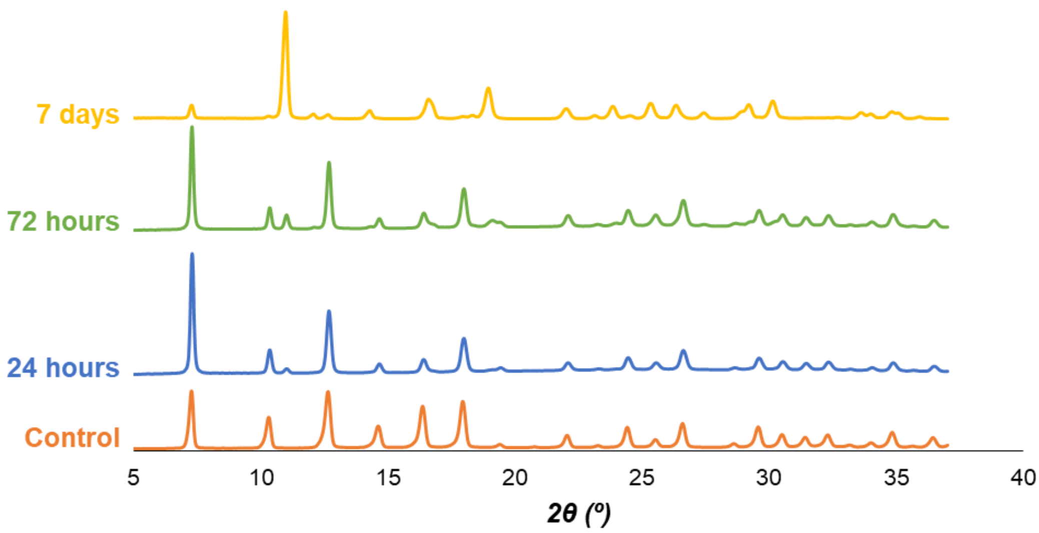

Figure S6. XRPD patterns of ZIF-8@HA composites in water at different scheduled times.

S4. Nutritional effect in wheat

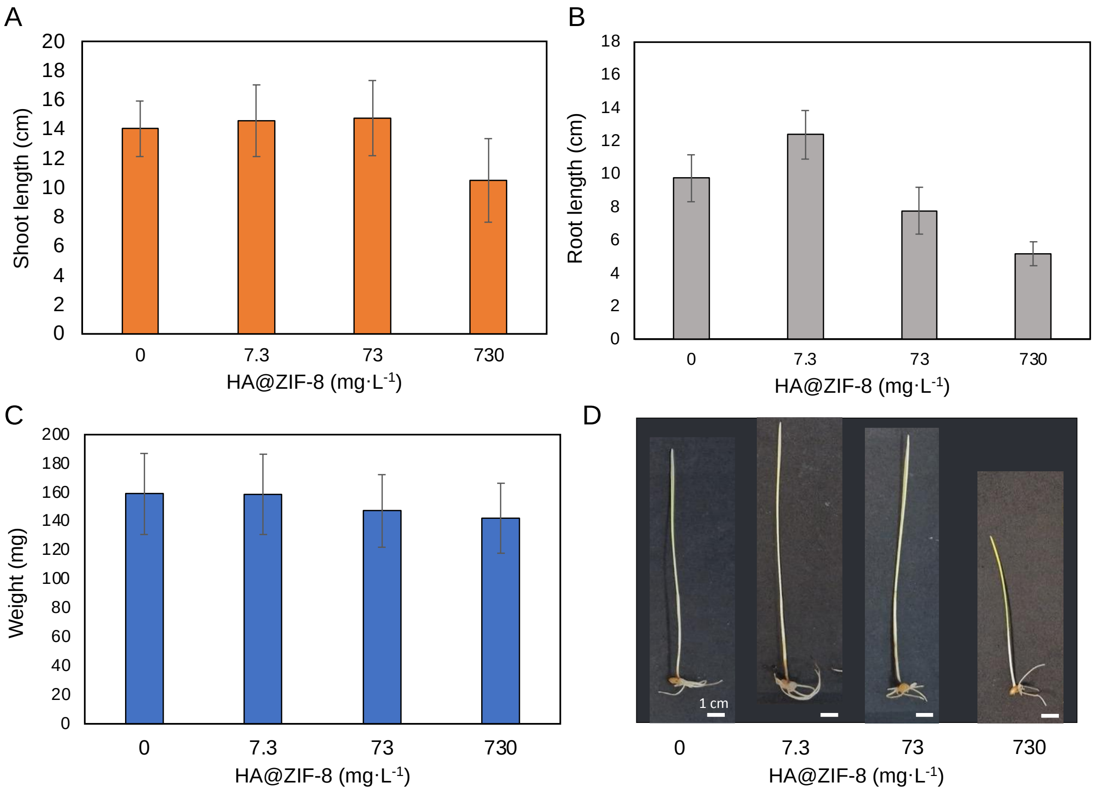

Figure S7. (A) Shoot length, (B) root length, (C) weight, and (D) images of seeds treated with incremental concentrations of ZIF-8@HA after 11 days. All parameters are given as averages with the corresponding standard errors (n=30).

S5. Antibacterial experiments

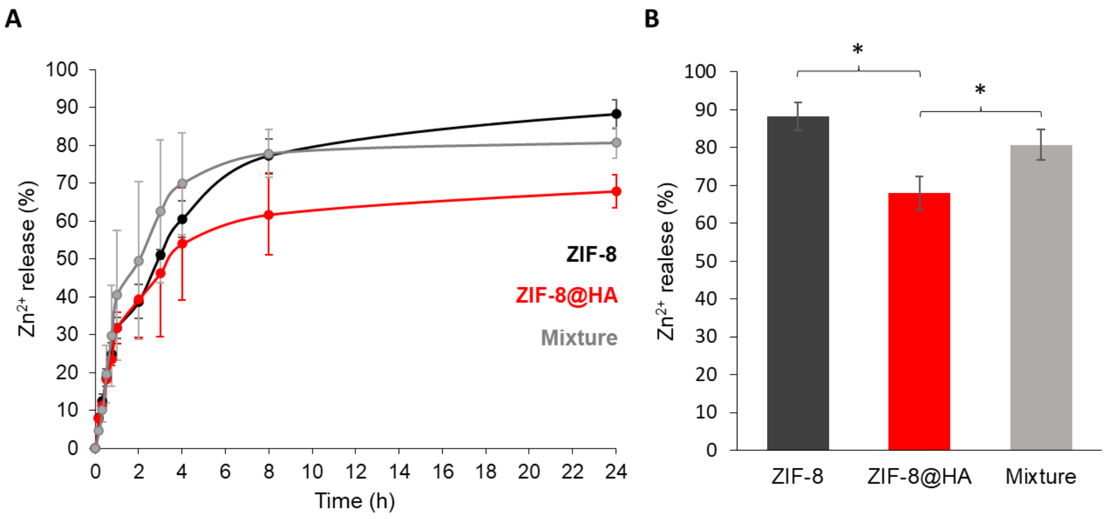

Figure S8. (A) Time-dependent Zn^{2+} release from ZIF-8, ZIF-8@HA, and mixture of both in KB media, and (B) total Zn^{2+} release at 24 hours. Experiments were performed in triplicate and the average and standard deviation are represented. The statistical analysis was performed using the one-way ANOVA where *p-value<0.05. Significant differences between ZIF-8@HA and the rest of samples were found at 24 h.

Table S1. Summary of the conditions employed to evaluate the growth inhibition of *Ps*.

Treatments	Conditions	Zn^{2+} (ppm)
Control	20 µl bacterial suspension + 80 µl KB + 100 µl H_2O	0
Zn^{2+}	20 µl bacterial suspension + 80 µl KB + 100 µl $ZnSO_4$ in H_2O	50
ZIF-8@HA	20 µl bacterial suspension + 180 µl of ZIF-8@HA in KB/H_2O (1:1 v/v)	50
ZIF-8	20 µl bacterial suspension + 180 µl of ZIF-8 in KB/H_2O (1:1 v/v)	50

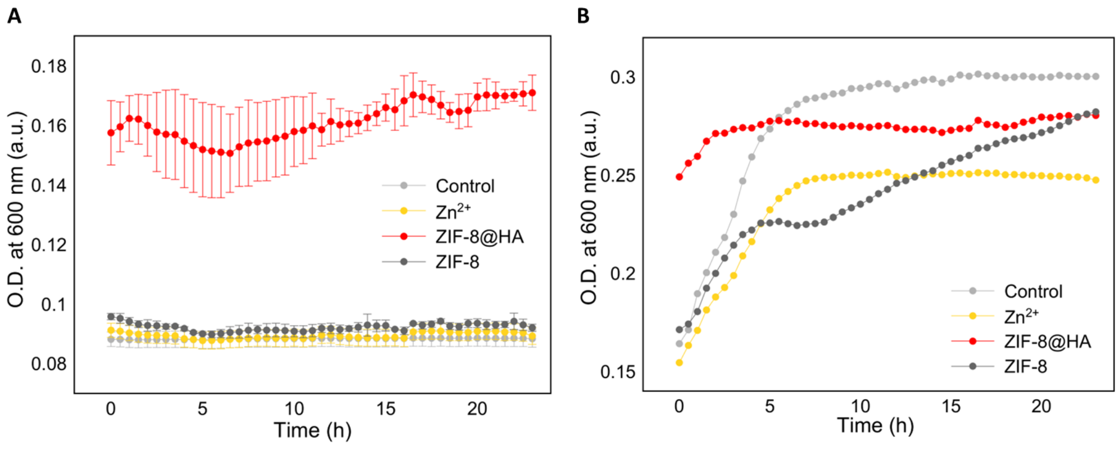

Figure S9. (A) Absorbance values of blank samples (non-inoculated with *Ps*). These constant values were subtracted to (B) the raw data of *Ps* growth curves, which were then plotted at Figure 6. Error bars were intentionally omitted in (B) to enhance clarity. All conditions (blank and inoculated samples) were performed in triplicates.
